# Long-Term Quality of Life and Functional Outcomes in Patients With Anorectal Malformations: A Retrospective Multicenter Study in Brussels, Belgium

**DOI:** 10.7759/cureus.107561

**Published:** 2026-04-23

**Authors:** Sandra Lopez Garri, Pierre Lingier, Martine Dassonville, Bérénice Tulelli

**Affiliations:** 1 Medical School, Unit of Pediatric Uro-visceral Surgery and Transplantation, Erasmus University Hospital, Université Libre de Bruxelles (ULB), Brussels, BEL; 2 Pediatric Surgery, Unit of Pediatric Uro-visceral Surgery and Transplantation, Erasmus University Hospital, Université Libre de Bruxelles (ULB) and Queen Fabiola Children's University Hospital (HUDERF), Brussels, BEL

**Keywords:** anorectal malformation, functional outcomes, hirschsprung's disease anorectal malformation quality of life questionnaire, long-term quality of life, quality of life

## Abstract

Introduction

Anorectal malformations (ARMs) are congenital conditions requiring surgical correction. Despite surgical intervention, patients often experience persistent fecal and urinary dysfunction, significantly impacting their quality of life (QoL). This multicenter retrospective study aimed to assess QoL in ARM patients, identify factors influencing QoL deterioration, and propose improved management strategies.

Methods

We retrospectively analyzed data from 39 ARM patients who underwent surgery between 1999 and 2019 at two Belgian hospitals. Patients completed the Hirschsprung's Disease Anorectal Malformation Quality of Life Questionnaire (HAQL). We also reviewed their medical records. Descriptive statistical analysis was performed.

Results

At a median follow-up of 106 months, functional outcomes showed high rates of constipation (87%), fecal incontinence (54%), and fecal soiling (87%). Fecal incontinence was more prevalent in patients with bulbar rectourethral and recto-vesical fistulas. Postoperative complications occurred in 72% of patients, primarily in those with high ARMs, and correlated with increased constipation and abdominal pain. Management gaps were observed: only 47% of constipated patients and 56% of incontinent patients underwent anorectal manometry, and only 50% of constipated patients utilized physiotherapy. QoL assessment revealed persistent digestive functional disorders and physical symptoms across age groups, which improved with age. However, emotional and body image issues persisted into adulthood.

Conclusion

Patients with ARMs frequently experience significant functional digestive disorders and impaired QoL. Suboptimal management, particularly regarding the use of anorectal manometry and physiotherapy, contributes to these challenges. A long-term, multidisciplinary follow-up is crucial. This follow-up should include systematic dietary support, perineal rehabilitation, and targeted interventions guided by anorectal manometry to improve QoL in this population.

## Introduction

Anorectal malformations (ARMs) represent a spectrum of complex congenital anomalies affecting the anus and rectum. These conditions arise from abnormal development during fetal life, leading to diverse anatomical presentations that require surgical correction. Despite significant advancements in surgical techniques over recent decades, patients with ARMs frequently experience persistent long-term functional sequelae. These include chronic fecal and urinary incontinence, intractable constipation, and recurrent abdominal pain. These enduring functional challenges extend beyond physical discomfort, profoundly impacting patients' overall quality of life (QoL). Consequently, effective management necessitates comprehensive, multidisciplinary care that addresses both the physical and psychosocial aspects of these conditions [1-3].

Assessing QoL in this specific patient population is crucial for understanding the true burden of ARM and evaluating treatment effectiveness. Validated instruments, such as the Hirschsprung's Disease Anorectal Malformation Quality of Life Questionnaire (HAQL), have been developed and are widely used to capture the multifaceted impact of ARM on daily life. While existing literature provides insights into short-to-medium term QoL outcomes, there remains a notable scarcity of data concerning the long-term QoL, particularly in adult ARM patients. This gap in knowledge limits our understanding of how these conditions evolve and affect individuals throughout their lifespan [2,4-7].

This multicenter retrospective study was designed to address this knowledge gap. We aimed to comprehensively evaluate the QoL of ARM patients who received treatment at two prominent Belgian hospitals. Specific objectives included identifying key factors that influence QoL, critically assessing the effectiveness of current management strategies employed in these institutions, and ultimately proposing evidence-based improvements for long-term patient care. A particular focus was placed on understanding QoL trajectories and management needs across different age groups, from childhood into adulthood.

## Materials and methods

Study design and patient population

**Figure 1:**
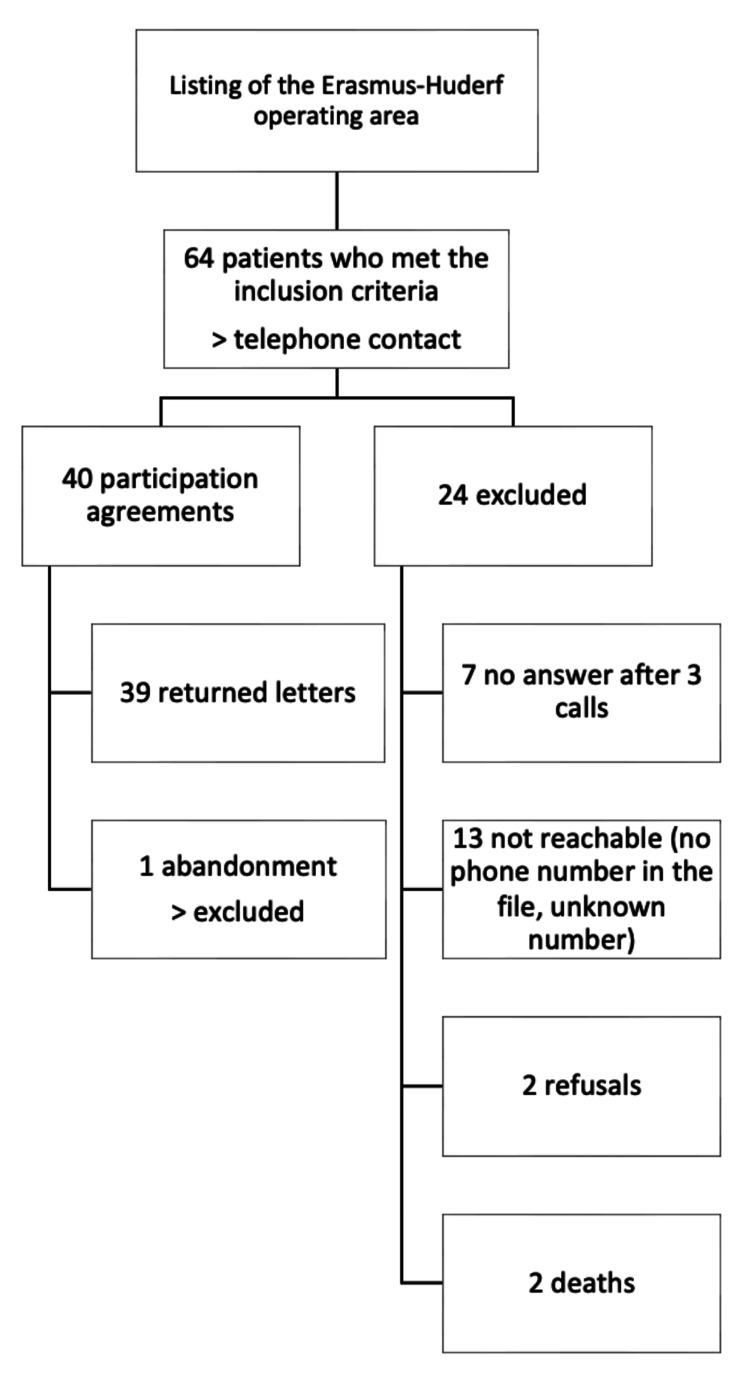
Patient selection criteria

The study was conducted at Erasmus Hospital and Queen Fabiola Children’s University Hospital in Brussels, Belgium, which are part of the same hospital network but represent two distinct sites. This study employed a multicenter, retrospective cohort design. We included patients who underwent surgical correction for ARMs between March 1999 and March 2019. This specific inclusion period ensured a minimum follow-up duration of four years between the primary surgical intervention and the administration of the QoL questionnaire. This interval was crucial for allowing sufficient time for postoperative functional stabilization and comprehensive QoL assessment. Consequently, patients operated on more recently were excluded to meet this minimum follow-up criterion. Additionally, patients who were unreachable, those who declined participation, and those who were deceased at the time of the study were excluded (Figure 1). Patients were systematically identified through a review of surgical databases at both participating institutions.

Data collection

Clinical data were extracted from the Global Medical Records (GMDs) of all included patients. A comprehensive set of variables was collected to provide a detailed clinical profile (Table 1). These variables encompassed the specific ARM type, a complete surgical history, and any postoperative complications, which were systematically classified according to the Clavien-Dindo system (Appendix 1) [8]. We also gathered information on the presence and management strategies for common functional issues, including constipation, fecal incontinence, fecal soiling, and abdominal pain. Furthermore, data on anorectal manometry results and the utilization of perineal rehabilitation physiotherapy were recorded.

**Table 1:** Full dataset ARM: Anorectal Malformation

Variables collected	
Gender	Age
Vaginal delivery	Cesarean section
Term	Birth weight
Birth size	Head circumference at birth
Apgar	Date of diagnosis
Type of ARM	Associated malformations
Follow-up hospital	Hospital of the intervention
Operator	Date of surgery
Operating times	Age at time of surgery
Type of surgery	Postoperative complication
Treatment of post-operative complications	Colostomy
Urinary incontinence	Fecal incontinence
Staining	Age of acquisition of fecal cleanliness
Constipation	Treatment of constipation
Diarrhea	Vomiting
Abdominal pain	Diet
Growth curve	Rectal manometry
Perineal physiotherapy	Psychological foloow-up
Schooling	Extraschool activity

QoL was a primary outcome measure and was assessed using the validated French version of the Hirschsprung's Disease Anorectal Malformation Quality of Life Questionnaire (HAQL) (Appendix 2) which was conducted with the approval of the ethics committee that authorized the study [5,7]. This comprehensive questionnaire is specifically designed for this patient population and evaluates QoL across multiple domains. It covers 10 core domains, with an additional 11th domain (sexuality) included for adult patients. These domains include aspects related to dietary habits (laxative and constipating diets), bowel function (diarrhea, constipation, fecal continence), urinary continence, physical symptoms, body image, social functioning, and emotional functioning. The HAQL is available in age-specific versions to ensure developmental appropriateness: eight to 11 years, 12 to 16 years, and ≥17 years. For very young children (under eight years), the eight to 11 years questionnaire was completed by their parents, serving as proxy reporters. All questionnaires were distributed to patients or their parents via postal mail [5].

Statistical analysis

Descriptive statistical analysis was performed using SPSS Statistics 2019 (IBM Corp., Armonk, NY, USA). Frequencies and percentages were used to summarize categorical data. Correlations between ARM type, post-operative complications, functional outcomes, and QoL domains were explored using only descriptive statistics in this study.

Ethical approval

The study protocol was reviewed and approved by the ethics committees of both Erasmus Hospital and Queen Fabiola Children's University Hospital (P2020/004; CCB:B406202042681).

## Results

Patient characteristics and post-operative complications

Initially, 64 patients met the preliminary eligibility criteria. Following a thorough screening process and after obtaining informed consent from either the patients or their legal guardians, 39 patients were ultimately included in the final analysis.

Among the 39 patients, the median age at the time of follow-up was nine years (range: 1-20 years). The median follow-up duration was 106 months (range: 12-250 months).

**Figure 2:**
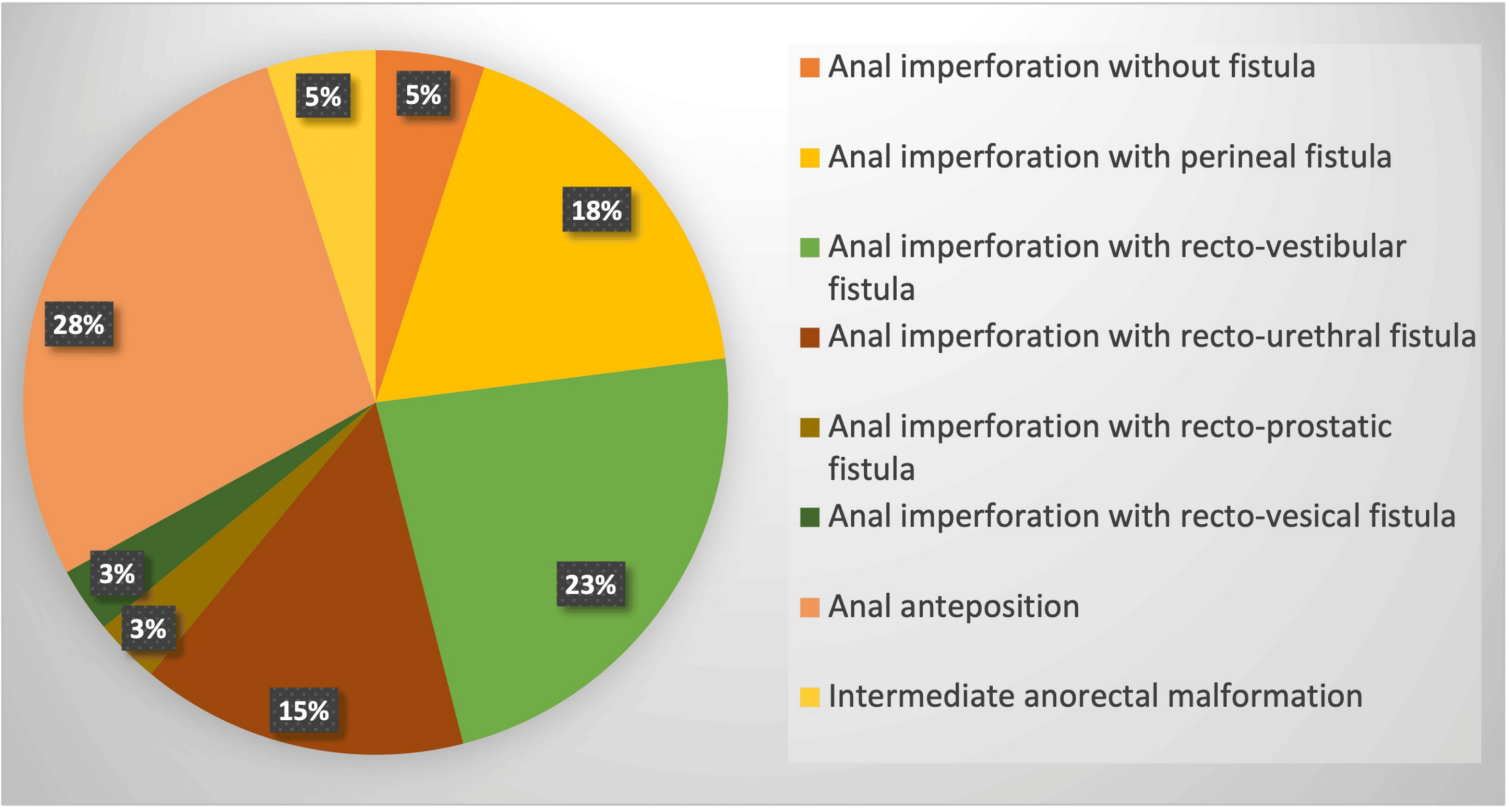
Distribution of the type of ARM in patients cohort ARM: Anorectal Malformation

The patient cohort exhibited a slight female predominance, with a sex ratio of 1.2 girls to one boy. The distribution of ARM types varied, as detailed in Figure 2. The most frequently observed ARM types involved anal imperforation with fistula, specifically recto-vestibular (23%), perineal (18%), and rectourethral (15%) malformations.

Postoperative complications were a significant finding, occurring in 74% (n=29) of the patient cohort (Table 2). According to the Clavien-Dindo classification, Grade II complications were the most prevalent, affecting 41% of patients. This was followed by Grade IIIb complications, observed in 33% of patients. Notably, patients with high ARMs demonstrated a higher frequency of both Grade II and Grade III complications, suggesting a correlation between ARM complexity and postoperative morbidity. Notably, the Melbourne classification (Appendix 3) is used to categorize ARMs into low, intermediate, or high types.

**Table 2:** Post-operative complications encountered according to the Clavien-Dindo classification "n" is the number of patients for the category mentioned

Post-operative complications according to Clavien-Dindo [8]	Type of complications
Grade I (n=9)	Infection of wound (n=3) - Ectropion of the anal mucosa (n=5) - Hypertrophic scar (n=1).
Grade II (n=16)	Dehiscence of wound (n=8) - onset of systemic infection requiring antibiotics (n=1) - irritated seat (n=15).
Grade IIIa (n=6)	Stenosis of colo-anal anastomosis requiring dilatation with Hégar candles under narcosis (n=6).
Grade IIIb (n=13)	Wound dehiscence requiring revision surgery (n=11) - rectal prolapse (n=1) - colostomy prolapse (n=1) - necrosis of stoma (n=1) - failure to restore colonic continuity (n=1) - peritonitis (n=1) - scarring hypertrophic requiring revision surgery (n=1).

Patient characteristics are summarized in Table 3.

**Table 3:** Description of the study population ARM: anorectal malformation, HUDERF: Queen Fabiola Children's University Hospital "n" is the number of patients for the category mentioned "%" is the percentage of patients represented in the category mentioned relative to the total number of patients in the sample (39 patients, unless otherwise stated). "N =" is the total number of patients for the category mentioned (when it was not 39)

Sample Characteristics	n (%)
Patients	39 (100)
Female	21 (54)
Male	18 (46)
Median age {range} in years	9 {1-20}
Under 8 years old	17 (43)
Girls under 8 years old	9
8-11 years old	12 (31)
Girls between 8 and 11 years old	9
12-16 years old	7 (18)
Girls between 12 and 16 years old	3
17 years or older	3 (8)
Girls 17 years or older	0
Born by spontaneous delivery	25 (69) N=36
Born at term	31 (84) N=37
Birth weight: Average {range} in grams	3207 {1940-4595} N=33
Anal imperforation without fistula	2 (5)
Perineal fistula	7 (18)
Recto-vestibular fistula	9 (23)
Recto-urethral fistula (bulbar)	6 (15)
Prostatic recto-urethral fistula	1 (3)
Recto-vesical fistula	1 (3)
Anal anteposition	11 (28)
ARM Intermediate	2 (5)
Associated malformation	17 (44)
1 type of associated malformation	6 (15)
2 types of associated malformation	1 (3)
3 types of associated malformation	6 (15)
4 types of associated malformation	3 (8)
5 types of associated malformation	1 (3)
Hospital of the intervention: Erasmus	17 (44)
Hospital of the intervention: HUDERF	22 (56)
Surgical interventions: 1 surgery	19 (49)
Surgical interventions: 2 surgeries	7 (18)
Surgical interventions: 3 surgeries	12 (31)
Surgical interventions: 4 or more surgeries	1 (2)
Post-operative complications according to the Clavien-Dindo classification [8]	28 (72)
Grade I	7 (18)
Grade II	14 (36)
Grade IIIa	8 (20)
Grade IIIb	13 (33)
Grade IV	0 (0)
Grade V	0 (0)
Colostomy	14 (36)
Urinary incontinence	6 (20) N=30
Age of acquisition of fecal cleanliness in years - Average {range}	4,3 {2,5-18} N=25
Fecal incontinence	16 (53) N=30
Fecal soiling	30 (86) N=35
Constipation	34 (87)
Medical treatment of constipation (Laxatives, glycerine suppositories, enemas, Hepar water)	34 (89) N=38
Vomiting	3 (8)
Abdominal pain	25 (64)
Diet	19 (49)
Normal growth curve	28 (76) N=37
Rectal manometry performed	18 (64) N=28
Physiotherapy for perineal rehabilitation	12 (31) N=35
Psychological follow-up required	8 (21)
Daycare/school	38 (97)

Functional digestive outcomes and management

Functional digestive disorders were highly prevalent within the study population (Table 4). Constipation was reported by a substantial 87% (n=34) of patients. Abdominal pain was reported in 64% of the patients in our cohort. Among the constipated individuals, 71% also experienced associated abdominal pain. Fecal incontinence affected 54% (n=21) of patients, while fecal soiling was reported by 87% (n=34). Both constipation and fecal soiling were observed across all types of ARMs. Fecal incontinence, however, showed a higher incidence in patients with bulbar rectourethral and recto-vesical fistulas, indicating a potential anatomical predisposition.

**Table 4:** Frequency of digestive disorders associates with ARM ARM: anorectal malformation

Digestive disorders	% of study patients
Constipation	87.20%
Diarrhea	28.20%
Abdominal pain	64.10%
Fecal incontinence	53.80%
Fecal soiling	87.20%

The management of these functional disorders revealed several areas for potential improvement. Although all constipated patients received some form of medical treatment, only 56% adhered to a specific dietary regimen. Diagnostic and therapeutic interventions were also underutilized. Anorectal manometry, a key diagnostic tool, was performed in only 47% of constipated patients and 56% of those experiencing fecal incontinence. Similarly, perineal rehabilitation physiotherapy, a common therapeutic approach, was utilized by only 50% of constipated patients and 56% of incontinent patients. These findings suggest a gap between the prevalence of functional issues and the consistent application of recommended management strategies.

**Figure 3:**
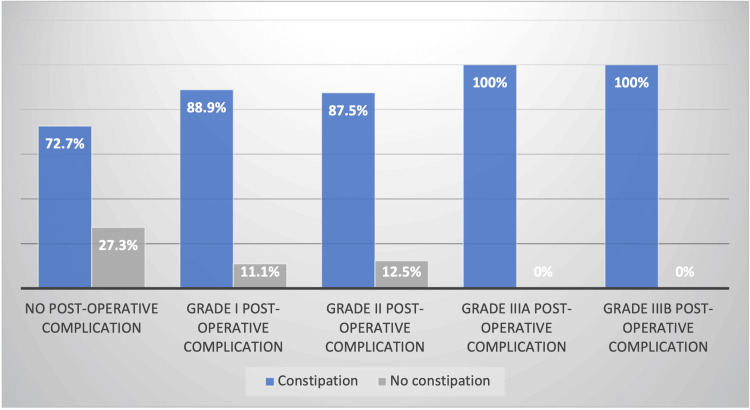
Influence of post-operative complications on constipation The information presented in this table represents original data from the Belgian multicenter cohort. Written informed consent was obtained from all participants.

**Figure 4:**
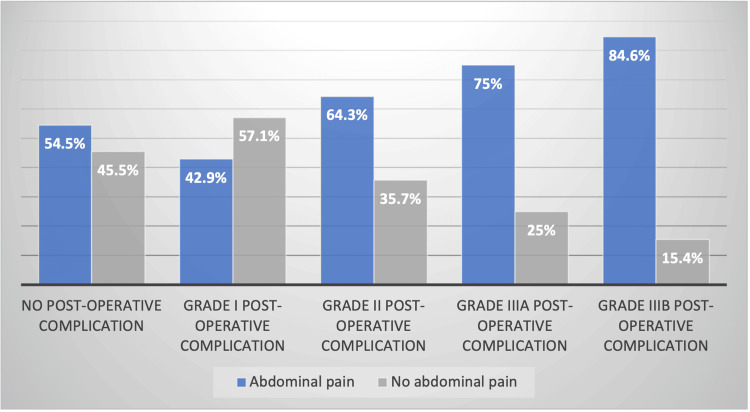
Influence of post-operative complications on abdominal pain The information presented in this table represents original data from the Belgian multicenter cohort. Written informed consent was obtained from all participants.

Constipation and abdominal pain, which are the two main complaints among patients operated on for ARM, were examined in greater detail. These two symptoms appear to be more prevalent in patients who experienced postoperative complications. This is illustrated by the data presented in Figure 3 and Figure 4.

Quality of life assessment (HAQL)

QoL questionnaire responses, gathered using the HAQL [5], were analyzed across three distinct age groups: children under eight years (n=17), adolescents aged eight to 16 years (n=16), and adults aged ≥17 years (n=3). It should be noted that, among the 39 patients included in the study, three returned their completed quality-of-life questionnaires after the prescribed deadline. Consequently, they could not be included in this part of the study.

Gastrointestinal and Physical Symptoms

Constipation and fecal continence disorders were consistently reported across all age groups. These issues were particularly pronounced in younger patients (under eight years and eight to 16 years). Physical symptoms, such as bloating and abdominal pain, were most prevalent in the eight-to-16-year age group. A general trend indicated that these gastrointestinal and physical symptoms tended to improve with increasing age, suggesting some adaptation or resolution over time.

Social Functioning

The assessment of social functioning revealed no impact in the adult age group. However, 24% of patients under eight years of age and 19% of those aged eight to 16 years do not participate in social activities (school, extracurricular activities). In addition, 53% of children under eight years and 33% of those aged eight to 12 years do not spend nights away from home. These findings suggest relatively impaired social integration in these age groups.

Emotional Functioning

Emotional well-being showed age-dependent variations. Patients aged 16 years or younger reported a higher emotional burden, frequently expressing feelings of shame, perceiving themselves as different from their peers, and experiencing teasing. While these negative emotional experiences tended to decrease with advancing age, the adult patient group still noted a persistent sense of uncertainty, highlighting ongoing psychological challenges.

Body Image

Dissatisfaction with body image was reported across all age groups but showed a distinct pattern. It was reported by 27% of patients under eight years, 13% of those aged eight to 16 years, and a significantly higher proportion of adult patients (67%). This suggests that body image concerns may become more prominent or re-emerge in adulthood.

Table 5 summarizes the most relevant findings regarding patients’ responses to the quality-of-life questionnaire.

**Table 5:** Key points derived from the quality-of-life questionnaires "N" represents the number of patients in each subgroup/the number of patients for whom a response was obtained through the questionnaire. "n" is the number of patients for the category mentioned "%" is the percentage of patients represented in the category mentioned relative to the total number of patients in the sample.

Responses to the quality of life questionnaire	Under 8 years (N=17)	8 - 16 years (N=16)	17 years and more (N=3)
	n (%)	n (%)	n (%)
Constipation	9 (53)	9 (56)	1 (33)
Fecal incontinence	4 (57) N=7	9 (56)	1 (33)
Abdominal pain	3 (20) N=15	7 (47) N=15	0 (0)
Does not engage in social activity	4 (24%)	2 (19)	0 (0)
Do not spend any nights away from home	9 (53)	5 (33) N=15	0 (0)
Feels a sense of shame	4 (29) N=14	3 (20) N=15	0 (0)
Feels different from others	4 (29) N=14	6 (40) N=15	1 (33)
Feels less attractive than others	1 (7) N=15	1 (6)	0 (0)
Feels dissatisfied with her/his body	4 (27) N=15	2 (13)	2 (67)

## Discussion

The present multicenter retrospective study provides compelling evidence of the significant and persistent functional digestive challenges faced by patients with ARMs. These challenges profoundly impact their overall QoL. Our findings demonstrate a high prevalence of constipation (87%), fecal incontinence (54%), and fecal soiling (87%). These rates are consistent with, or in some instances slightly higher than, those reported in other international cohorts [1,9,10]. This potentially elevated prevalence in the present study may be attributable to the high proportion of complex ARM types within the patient population and the identified gaps in current management strategies.

A crucial finding is the clear correlation between higher-grade postoperative complications and increased rates of chronic constipation and abdominal pain. This underscores the profound and long-lasting impact that initial surgical outcomes can have on a patient's QoL, extending far beyond the immediate recovery period. Furthermore, data reveal a suboptimal utilization of essential diagnostic tools, such as anorectal manometry, and subsequent targeted interventions, including perineal rehabilitation physiotherapy. The underuse of anorectal manometry is particularly concerning, as it is a critical tool for accurately identifying the underlying physiological mechanisms of functional disorders. This diagnostic clarity is essential for tailoring specific therapies. For instance, physiotherapy is known to be highly effective, with reported success rates ranging from 50-85%[11] in appropriate cases. Similarly, the lack of rigorous and systematic dietary follow-up represents a missed opportunity to address constipation and abdominal pain, which were highly prevalent in the cohort and significantly contribute to patient distress.

QoL assessment, utilizing the HAQL questionnaire, revealed distinct age-dependent trends. While gastrointestinal and physical symptoms showed a tendency to improve with increasing age, emotional and body image concerns proved to be more enduring, persisting significantly into adulthood [12,13]. This observation strongly aligns with previous studies that highlight the substantial psychological burden associated with ARM. It emphasizes the critical need for systematic psychological support, particularly during the vulnerable transition from adolescence to adulthood, to proactively address issues of self-confidence, body dissatisfaction, and social integration [12,14]. Interestingly, our finding that social functioning was not significantly impacted across age groups differs from some existing literature [6,15]. This discrepancy might be influenced by the specific characteristics of our patient population, cultural factors, or potentially the sensitivity of the HAQL questionnaire in capturing subtle social challenges within this domain.

Based on these findings, we strongly advocate for the implementation of a more structured, comprehensive, and long-term multidisciplinary follow-up program for all ARM patients. This integrated care model should systematically include regular intestinal management consultations involving a specialized team comprising surgeons, gastroenterologists, dieticians, and psychologists. Such a holistic approach is vital not only for optimizing the management of functional disorders but also for enhancing overall QoL and providing continuous support to patients as they navigate various developmental stages.

Limitations

This study is subject to several limitations inherent to its retrospective design. The reliance on historical medical records inherently limits control over data completeness and introduces potential for information bias. Furthermore, the retrospective nature of QoL questionnaire administration may introduce recall bias, potentially affecting the accuracy of patient-reported outcomes. The relatively small sample size, particularly the very limited number of adult patients (n=3), significantly restricts the generalizability of our long-term QoL findings to the broader ARM population. Furthermore, there may be a selection bias, as only 61% of all eligible patients were ultimately included in the study. Additionally, QoL data for younger children were obtained through parent-proxy reports, which, while valuable, may not always fully capture the child's subjective emotional experience or perception of their condition.

Future research

To build upon these preliminary findings, future prospective studies with substantially larger cohorts are warranted. A particular emphasis should be placed on recruiting a more robust sample of adult ARM patients to provide more comprehensive and reliable long-term QoL data. Longitudinal studies, designed to track the same patients over extended periods, would offer invaluable insights into the evolving challenges associated with ARM and the long-term effectiveness of various interventions. Moreover, future research efforts should focus on the rigorous development, implementation, and evaluation of standardized multidisciplinary care pathways.

## Conclusions

Despite continuous advancements in surgical techniques for ARMs, the present study confirms that patients frequently endure persistent functional digestive disorders. These disorders, including chronic constipation, fecal incontinence, and soiling, significantly impair their overall QoL. Our findings underscore the critical necessity for a robust, long-term, and truly multidisciplinary follow-up approach. This comprehensive care model must integrate systematic dietary support, targeted perineal rehabilitation guided by precise findings from anorectal manometry, and essential psychological support. Implementing these integrated care strategies is not merely beneficial but essential. It will optimize functional outcomes, mitigate long-term complications, and substantially enhance the overall QoL for individuals living with ARMs across their lifespan.

## Appendices

Appendix 1: Clavien-Dindo classification

**Table 6:** Classification of Clavien-Dindo [8]

Grade	Grade Description
Grade I	Any deviation from the normal course of the operation without requiring pharmacological treatment or surgical, endoscopic and radiological interventions. Acceptable medical treatments include: antiemetics, antipyretics, analgesics, diuretics, electrolytes and physical therapy. This category also includes open wound infections.
Grade II	Complication requiring pharmacological treatment with drugs other than those approved for Grade I complications. Blood transfusions and total parenteral nutrition are also included.
Grade III	Complication requiring surgical, endoscopic or radiological intervention
IIIa	Procedure without general anesthesia
IIIb	Procedure under general anesthesia
Grade IV	Life-threatening complication requiring intensive care management.
IVa	Single organ dysfunction (including dialysis)
IVb	Multiple organ dysfunction
Grade V	Death of the patient
Suffixe "d"	If the patient has a complication at the time of discharge, the suffix "d" is added to the corresponding complication level. This indicates the need for follow-up.

Appendix 2: Hirschsprung's Disease Anorectal Malformation Quality of Life Questionnaire (HAQL)

**Table 7:** Quality of Life Questionnaire for Parents [5]

Quality of Life Questionnaire for Parents
Below you will find a questionnaire that you, as a parent, are asked to complete. This allows us to gain an understanding of the quality of life of a child who has undergone surgery due to an anorectal malformation.
A. The gastrointestinal system
Laxative diet
Does your child eat certain foods that could soften his/her stools?
YES – NO
Does your child avoid certain foods that could harden his/her stools?
YES – NO
Constipating diet
Does your child eat certain foods that could harden his/her stools?
YES – NO
Does your child avoid certain foods that could soften his/her stools?
YES – NO
Does your child experience diarrhea at certain times?
Less than 4 times per day?
YES – NO
More than 4 times per day?
YES – NO
Does your child have firm and hard stools (constipation)?
YES – NO
Fecal incontinence
Is your child always alert and attentive to the presence of a toilet in his/her immediate surroundings?
YES – NO
Does your child experience stool leakage before reaching the toilet?
YES – NO
Does your child have soiled underwear during the day?
YES – NO
Does your child have soiled underwear during the night?
YES – NO
Does your child experience stool leakage during the night?
YES – NO
Does your child experience stool leakage during physical activity?
YES – NO
Does your child experience stool leakage during emotional situations?
YES – NO
Does your child experience stool leakage when coughing or sneezing?
YES – NO
Urinary incontinence
Does your child experience urine leakage before reaching the toilet?
YES – NO
Does your child experience urine leakage during physical activity?
YES – NO
Does your child experience urine leakage during emotional situations?
YES – NO
Does your child experience urine leakage when coughing or sneezing?
YES – NO
B. Physical symptoms
Does your child often experience a bloated feeling?
YES – NO
Does your child feel the urge to go to the toilet when his/her stomach is full?
YES – NO
Does your child feel the urge to go to the toilet when his/her stomach is not full?
YES – NO
Does your child experience difficulties during bowel movements?
YES – NO
Does your child have difficulty distinguishing between passing gas and having a bowel movement?
YES – NO
Does your child often experience abdominal pain?
YES – NO
Does your child have bowel movements?
YES – NO
C. Social functioning
Does your child engage in daily activities (school, work, ...)?
YES – NO
Does your child participate in social activities (sports, hobbies, ...)?
YES – NO
Does your child sometimes stay overnight away from home (family, friends, ...)?
YES – NO
If your child is between 12 and 16 years old, please also answer the following questions:
Does your child often experience unpleasant, bitter, or sad feelings?
YES – NO
Does your child desire sexual experiences?
YES – NO – I do not know
D. Emotional functioning
Is your child often ashamed of himself/herself?
YES – NO
Does your child feel different from others?
YES – NO
Does your child feel less valued by others?
YES – NO
Is your child worried or afraid that others can smell his/her stool?
YES – NO
If your child is between 8 and 16 years old, please also answer the following questions:
Does your child feel ashamed when leaving the classroom to go to the toilet?
YES – NO
Does your child experience more bullying than other children?
YES – NO
E. Self-image
Does your child feel less attractive than others?
YES – NO
Is your child dissatisfied with his/her own body?
YES – NO

**Table 8:** Quality of Life Questionnaire for Children under 8 years old [5]

Quality of life Questionnaire for children under 8 years old
A. Gastrointestinal system
Laxative diet
Do you eat special food to make your bowel movements easier?
YES – NO
Do you avoid food that makes your stools harder?
YES – NO
Constipating diet
Do you eat special food to make your stools firmer?
YES – NO
Do you avoid certain foods to soften your stools?
YES – NO
Do you suffer from diarrhea?
Less than 4 times per day?
YES – NO
4 times per day or more?
YES – NO
Do you have hard stools (constipation)?
YES – NO
Fecal incontinence disorder (loss of stool).
Do you check in advance whether there is a toilet nearby?
YES – NO
Do you have stool leakage before going to the toilet?
YES – NO
Do you regularly have stool leakage during the day (soiled underwear)?
YES – NO
Do you have stool leakage at night (soiled underwear)?
YES – NO
Do you have stool leakage during sports?
YES – NO
Do you have stool leakage during emotional moments?
YES – NO
Do you have stool leakage when sneezing or coughing?
YES – NO
Urinary incontinence conditions
Do you have urine leakage before going to the toilet?
YES – NO
Do you have urine leakage during sports?
YES – NO
Do you have urine leakage during emotional moments?
YES – NO
Do you have urine leakage when sneezing or coughing?
YES – NO
B. Physical symptoms
Do you often feel bloated?
YES – NO
Do you often feel the need to go to the toilet to empty your bowels?
YES – NO
Do you sometimes feel that you need to go to the toilet, but nothing comes out?
YES – NO
Do you have problems with your bowel movements?
YES – NO
Do you have difficulty when you cannot distinguish between passing gas and having a bowel movement?
YES – NO
Do you regularly have stomach pain?
YES – NO
Do you have bowel movements?
YES – NO
C. Social functioning
Do you have daily activities (school, work, ...)?
YES – NO
Do you have social activities (sports, hobbies, ...)?
YES – NO
Do you sometimes spend the night away from home? (friends, family, work, ...)?
YES – NO
D. Emotional functioning
Do you sometimes feel ashamed?
YES – NO
Do you feel different from others?
YES – NO
Do you feel less valued by others?
YES – NO
Are you afraid that others can smell your stool?
YES – NO
E. Body image
Do you feel less attractive than others?
YES – NO
Are you dissatisfied with your body?
YES – NO

**Table 9:** Quality of Life Questionnaire for Children between 8 and 11 years old [5]

Quality of Life Questionnaire for Children between 8 and 11 years old
Do not hesitate to ask your parents for help if there is something you do not understand, but remember that you are the one who must answer the questions according to what you do/feel!
A. Gastrointestinal system
Laxative diet
Do you eat special foods to soften your stools?
YES – NO
Do you avoid eating foods that make your stools harder?
YES – NO
Constipating diet
Do you eat special foods to harden your stools?
YES – NO
Do you avoid eating foods that could soften your stools?
YES – NO
Do you have episodes of diarrhea?
Less than 4 episodes per day?
YES – NO
4 episodes or more per day?
YES – NO
Do you have firm and hard stools (constipation)?
YES – NO
Fecal continence disorder
Are you always attentive to whether there is a toilet nearby where you are?
YES – NO
Do you have stool leakage before reaching the toilet?
YES – NO
Do you have stains in your underwear during the day?
YES – NO
Do you have nighttime stains in your underwear?
YES – NO
Do you have stool leakage during the night?
YES – NO
Do you have stool leakage during physical activity?
YES – NO
Do you have stool leakage during emotional moments?
YES – NO
Do you have stool leakage when coughing or sneezing?
YES – NO
Urinary continence disorders
Do you have urine leakage before going to the toilet?
YES – NO
Do you have urine leakage during physical activity?
YES – NO
Do you have urine leakage during emotional moments?
YES – NO
Do you have urine leakage when coughing or sneezing?
YES – NO
B. Physical symptoms
Do you often feel bloated?
YES – NO
Do you feel the need to have a bowel movement when your intestines are full of stool?
YES – NO
Do you sometimes have to go to the toilet even though you do not feel the need?
YES – NO
Do you have difficulties having a bowel movement?
YES – NO
Do you have difficulty distinguishing between passing gas and having a bowel movement?
YES – NO
Do you regularly have abdominal pain?
YES – NO
Do you have bowel movements?
YES – NO
C. Social functioning
Do you have daily activities (school, job, ...)?
YES – NO
Do you have a social activity (sports, hobbies, ...)?
YES – NO
Do you sometimes spend the night away from home (friends, family, work, ...)?
YES – NO
D. Emotional functioning
Do you often feel a sense of shame about yourself?
YES – NO
Do you feel different from others?
YES – NO
Do you feel less appreciated by others?
YES – NO
Are you afraid that others can smell your stool?
YES – NO
If you are under 8 years old, go directly to the next section “Body image”. If you are 8 years old or older, you can answer the following 2 questions:
Are you ashamed to leave your classroom to go to the toilet?
YES – NO
Do you feel that you are teased more than other children?
YES – NO
E. Body image
Do you feel less attractive than others?
YES – NO
Do you feel dissatisfied with your body?
YES – NO

**Table 10:** Quality of Life Questionnaire for Children between 12 and 16 years old [5]

Quality of Life Questionnaire for Children between 12 and 16 years old
Do not hesitate to ask your parents for help if there is something you do not understand but remember that you are the one who must answer the questions according to what you do/feel!
A. Gastrointestinal system
Laxative diet
Do you eat special foods to soften your stools?
YES – NO
Do you avoid eating foods that make your stools harder?
YES – NO
Constipating diet
Do you eat special foods to harden your stools?
YES – NO
Do you avoid eating foods that could soften your stools?
YES – NO
Do you have episodes of diarrhea?
Less than 4 episodes per day?
YES – NO
4 episodes or more per day?
YES – NO
Do you have firm and hard stools (constipation)?
YES – NO
Fecal continence disorder
Are you always attentive to whether there is a toilet nearby where you are?
YES – NO
Do you have stool leakage before reaching the toilet?
YES – NO
Do you have stains in your underwear during the day?
YES – NO
Do you have nighttime stains in your underwear?
YES – NO
Do you have stool leakage during the night?
YES – NO
Do you have stool leakage during physical activity?
YES – NO
Do you have stool leakage during emotional moments?
YES – NO
Do you have stool leakage when coughing or sneezing?
YES – NO
Urinary continence disorders
Do you have urine leakage before going to the toilet?
YES – NO
Do you have urine leakage during physical activity?
YES – NO
Do you have urine leakage during emotional moments?
YES – NO
Do you have urine leakage when coughing or sneezing?
YES – NO
B. Physical symptoms
Do you often feel bloated?
YES – NO
Do you feel the need to have a bowel movement when your intestines are full of stool?
YES – NO
Do you sometimes have to go to the toilet even though you do not feel the need?
YES – NO
Do you have difficulties having a bowel movement?
YES – NO
Do you have difficulty distinguishing between passing gas and having a bowel movement?
YES – NO
Do you regularly have abdominal pain?
YES – NO
Do you have bowel movements?
YES – NO
C. Social functioning
Do you have daily activities (school, job, ...)?
YES – NO
Do you have a social activity (sports, hobbies, ...)?
YES – NO
Do you sometimes spend the night away from home (friends, family, work, ...)?
YES – NO
Do you often feel bitterness?
YES – NO
Do you fantasize about having sexual relations?
YES – NO
D. Emotional functioning
Do you often feel a sense of shame about yourself?
YES – NO
Do you feel different from others?
YES – NO
Do you feel less appreciated by others?
YES – NO
Are you afraid that others can smell your stool?
YES – NO
Are you ashamed to leave your classroom to go to the toilet?
YES – NO
Do you feel that you are teased more than other children?
YES – NO
E. Body image
Do you feel less attractive than others?
YES – NO
Do you feel dissatisfied with your body?
YES – NO

**Table 11:** Quality of Life Questionnaire for Children of 17 years and older [5]

Quality of Life Questionnaire for Children of 17 years and older
A. Gastrointestinal system
Laxative diet
Do you avoid eating foods that harden your stools?
YES – NO
Constipating diet
Do you eat special foods to harden your stools?
YES – NO
Do you avoid eating foods that could soften your stools?
YES – NO
Do you have episodes of diarrhea?
Less than 4 episodes per day?
4 episodes or more per day?
YES – NO
Do you have solid and hard stools (constipation)?
YES – NO
Fecal continence disorder
Are you always careful to make sure there is always a toilet nearby wherever you are?
YES – NO
Do you have stool leakage before reaching the toilet?
YES – NO
Do you have soiling in your underwear during the day?
YES – NO
Do you have nighttime soiling in your underwear?
YES – NO
Do you have stool leakage during the night?
YES – NO
Do you have stool leakage during physical activity?
YES – NO
Do you have stool leakage during emotional moments?
YES – NO
Do you have stool leakage when coughing or sneezing?
YES – NO
Urinary continence disorders
Do you have urine leakage before going to the toilet?
YES – NO
Do you have urine leakage during physical activity?
YES – NO
Do you have urine leakage during emotional moments?
YES – NO
Do you have urine leakage when coughing or sneezing?
YES – NO
B. Physical symptoms
Do you often feel bloated?
YES – NO
Do you feel the need to have a bowel movement when your intestines are full of stool?
YES – NO
Do you have to have a bowel movement even when you do not feel the need?
YES – NO
Do you have difficulty having a bowel movement?
YES – NO
Do you have difficulty distinguishing between gas and stool?
YES – NO
Do you regularly have abdominal pain?
YES – NO
Do you have bowel movements?
YES – NO
C. Social functioning
Do you have daily activities (school, job, …)?
YES – NO
Do you have a social activity (sports, various leisure activities, …)?
YES – NO
Do you sometimes spend the night away from home (friends, family, work, …)?
YES – NO
D. Emotional functioning
Do you often feel a sense of shame about yourself?
YES – NO
Do you feel different from others?
YES – NO
Do you feel less appreciated by others?
YES – NO
Are you afraid that others might smell your stool?
YES – NO
Do you regularly feel embarrassed?
YES – NO
Do you regularly feel a sense of uncertainty?
YES – NO
Do you feel worried about the future?
YES – NO
E. Body image
Do you feel less attractive than others?
YES – NO
Do you feel dissatisfied with your body?
YES – NO
F. Sexual function
Are you interested in sex?
YES – NO
Do you have an active sex life?
YES – NO

Appendix 3: Melbourne classification

**Table 12:** Classification of Melbourne (Girls) [16] ARM: Anorectal Malformation

Higher ARMs	Intermediate ARMS	Lower ARMs
Anorectal agenesis without fistula	Anal agenesis without fistula	Complete anal membrane
Anorectal agenesis with rectovesical fistula	Anal agenesis with rectovaginal fistula	Anal stenosis
Anorectal agenesis with rectovaginal fistula	Anal agenesis with rectovestibular fistula	Anteriorly displaced anus
Anorectal agenesis with rectocloacal fistula	Anorectal stenosis	Anocutaneous fistula
Rectal atresia		Vulvar anus
		Anovulvar fistula

**Table 13:** Classification of Melbourne (Boys) [16] ARM: Anorectal Malformation

Higher ARMs	Intermediate ARMS	Lower ARMs
Anorectal agenesis without fistula	Anal agenesis without fistula	Complete anal membrane
Anorectal agenesis with rectovesical fistula	Anal agenesis with rectobulbar fistula	Anal stenosis
Anorectal agenesis with rectoprostatic urethral fistula	Anorectal stenosis	Anteriorly displaced anus
Rectal atresia		Anocutaneous fistula

## References

[1] Crétolle C, Rousseau V, Lottmann H (2014). Reconnaître les malformations anorectales: du diagnostic à la transition vers l’adulte [Recognizing anorectal malformations: from diagnosis to transition to adulthood]. Hepato-Gastro Oncol Dig.

[2] Rintala RJ, Pakarinen MP (2010). Outcome of anorectal malformations and Hirschsprung's disease beyond childhood. Semin Pediatr Surg.

[3] Drissi F, Wyart V, Lehur PA (2015). Séquelles digestives et psychosociales à l’âge adulte de la maladie de Hirschsprung et des malformations anorectales [Digestive and psychosocial sequelae in adulthood of Hirschsprung disease and anorectal malformations]. Colon Rectum.

[4] Grano C, Aminoff D, Lucidi F, Violani C (2012). Long-term disease-specific quality of life in children and adolescent patients with ARM. J Pediatr Surg.

[5] Baayen C, Feuillet F, Clermidi P, Crétolle C, Sarnacki S, Podevin G, Hardouin JB (2017). Validation of the French versions of the Hirschsprung's disease and anorectal malformations Quality of Life (HAQL) questionnaires for adolescents and adults. Health Qual Life Outcomes.

[6] Hanneman MJ, Sprangers MA, De Mik EL (2001). Quality of life in patients with anorectal malformation or Hirschsprung's disease: development of a disease-specific questionnaire. Dis Colon Rectum.

[7] Clermidi P, Podevin G, Crétolle C, Sarnacki S, Hardouin JB (2013). The challenge of measuring quality of life in children with Hirschsprung's disease or anorectal malformation. J Pediatr Surg.

[8] Dindo D, Demartines N, Clavien PA (2004). Classification of surgical complications: a new proposal with evaluation in a cohort of 6336 patients and results of a survey. Ann Surg.

[9] Schmiedeke E, Zwink N, Schwarzer N (2012). Unexpected results of a nationwide, treatment-independent assessment of fecal incontinence in patients with anorectal anomalies. Pediatr Surg Int.

[10] Langemeijer RA, Molenaar JC (1991). Continence after posterior sagittal anorectoplasty. J Pediatr Surg.

[11] Valancogne G (2009). Aspects spécifiques de la rééducation ano-rectale de l'enfant [Specific aspects of anorectal rehabilitation in children]. Kinésithér Sci.

[12] Grano C, Bucci S, Aminoff D, Lucidi F, Violani C (2015). Transition from childhood to adolescence: quality of life changes 6 years later in patients born with anorectal malformations. Pediatr Surg Int.

[13] Wigander H, Nisell M, Frenckner B, Wester T, Brodin U, Öjmyr-Joelsson M (2019). Quality of life and functional outcome in Swedish children with low anorectal malformations: a follow-up study. Pediatr Surg Int.

[14] Grano C, Aminoff D, Lucidi F, Violani C (2010). Disease-specific quality of life in children and adults with anorectal malformations. Pediatr Surg Int.

[15] Feng X, Lacher M, Quitmann J, Witt S, Witvliet MJ, Mayer S (2020). Health-related quality of life and psychosocial morbidity in anorectal malformation and Hirschsprung’s disease. Eur J Pediatr Surg.

[16] (2015). Découverte d’une malformation anorectale en salle de travail: et la suite? [Diagnosis of an anorectal malformation in the delivery room: what next?]. https://www.realites-pediatriques.com/decouverte-dune-malformation-anorectale-en-salle-de-travail%e2%80%89-et-%e2%80%afla%e2%80%afsuite%e2%80%89/.

